# An Uncommon Presentation of Selpercatinib (Retevmo)-Induced Severe Transaminitis: A Biopsy-Proven Case Report

**DOI:** 10.7759/cureus.105372

**Published:** 2026-03-17

**Authors:** Navanita Biswas, Krishi Dudhia

**Affiliations:** 1 Internal Medicine, Tower Health Reading Hospital, Reading, USA; 2 Medicine, Drexel University College of Medicine, Philadelphia, USA

**Keywords:** drug-induced hepatotoxicity (dih), liver biopsy, retevmo, ret inhibitor, selpercatinib

## Abstract

Selpercatinib (Retevmo) is a selective rearranged during transfection (RET) kinase inhibitor approved for RET-altered malignancies, including thyroid carcinoma. Hepatic enzyme elevations have been reported; however, biopsy-proven drug-induced liver injury (DILI) associated with selpercatinib is uncommon. We report a case of a 59-year-old woman with recurrent papillary thyroid carcinoma who developed progressive gastrointestinal symptoms followed by severe hepatocellular transaminitis approximately four weeks after initiating selpercatinib (160 mg twice daily). Laboratory evaluation demonstrated marked aminotransferase elevation (aspartate aminotransferase: 525 U/L and alanine aminotransferase: 639 U/L) with mild hyperbilirubinemia (total bilirubin: 2.1 mg/dL), and aminotransferases peaked above 800 U/L during hospitalization. Comprehensive workup for alternative etiologies, including viral hepatitis and autoimmune hepatitis (negative antinuclear antibody and smooth muscle antibody with normal immunoglobulin levels), was unrevealing, and abdominal imaging showed no acute abnormalities. Liver biopsy demonstrated sparse portal lymphocytic inflammation with intact bile ducts and prominent centrilobular (zone 3) hepatocellular necrosis accompanied by macrophages and eosinophils, consistent with DILI. Selpercatinib was discontinued, resulting in progressive biochemical improvement with aminotransferases declining to <100 U/L within approximately four months. Given oncologic necessity, selpercatinib was reintroduced at a reduced dose (40 mg daily) without recurrence of severe transaminitis. This case highlights that selpercatinib can cause clinically significant hepatocellular DILI and underscores the diagnostic value of liver biopsy in distinguishing DILI from autoimmune hepatitis, particularly when considering safe dose modification and rechallenge.

## Introduction

Selpercatinib is a highly selective rearranged during transfection (RET) kinase inhibitor used in RET-mutated or RET-fusion-positive malignancies [1]. In clinical trials, selpercatinib demonstrated significant antitumor activity with an overall favorable safety profile [2]. However, elevations in serum aminotransferases have been reported, with grade 3-4 transaminase elevations occurring in a minority of treated patients. Serious hepatic adverse reactions have been reported in approximately 3% of patients receiving selpercatinib in clinical trials, including the LIBRETTO-001 study, highlighting hepatotoxicity as a clinically relevant but relatively uncommon complication of therapy [2].

The precise mechanism underlying selpercatinib-associated hepatotoxicity remains incompletely understood. The pattern of aminotransferase elevation and improvement following dose interruption or reduction suggests a component of dose-related direct hepatocellular injury rather than an autoimmune-mediated process. Selpercatinib undergoes hepatic metabolism predominantly through the cytochrome P450 system, particularly *CYP3A4*. As a result, variations in metabolic activity or concomitant use of *CYP3A4* inhibitors or inducers may alter drug exposure and potentially increase susceptibility to liver injury [1].

Drug-induced liver injury (DILI) remains a diagnosis of exclusion and can mimic autoimmune or immune-mediated hepatitis, particularly in oncology patients. Histologically, DILI can demonstrate a wide spectrum of morphological patterns depending on the mechanism and severity of injury. Common patterns include necroinflammatory (hepatocellular) injury, cholestatic injury, vascular injury, and steatosis or steatohepatitis-like changes. Recognition of these patterns can help differentiate DILI from other causes of liver dysfunction and guide clinical interpretation of biopsy findings [3]. We present a case of selpercatinib-induced hepatotoxicity confirmed by liver biopsy, with prominent centrilobular necrosis. The objective of this report is to describe a biopsy-proven case of selpercatinib-induced hepatotoxicity presenting with severe transaminitis and centrilobular necrosis, highlighting the clinicopathologic features and emphasizing the importance of early recognition and diagnostic evaluation of this rare adverse effect.

## Case presentation

A 59-year-old woman with a history of papillary thyroid carcinoma, initially diagnosed in 2017 and treated with total thyroidectomy followed by radioactive iodine therapy on two occasions, later developed disease recurrence and was started on oral selpercatinib (Retevmo) 160 mg twice daily for recurrent disease.

Approximately four weeks after initiation of selpercatinib, the patient developed progressive gastrointestinal symptoms, including nausea, diarrhea, and poor oral intake, which persisted for approximately two weeks. These symptoms were accompanied by generalized weakness, fatigue, and intermittent right upper quadrant abdominal pain. The abdominal pain was described as sharp and stabbing, non-radiating, intermittent, and self-resolving without specific intervention. 

She additionally endorsed chronic intermittent xerostomia, which she reported had been present since her initial radioactive iodine treatment. She denied alcohol use and reported no significant acetaminophen use.

**Figure 1:**
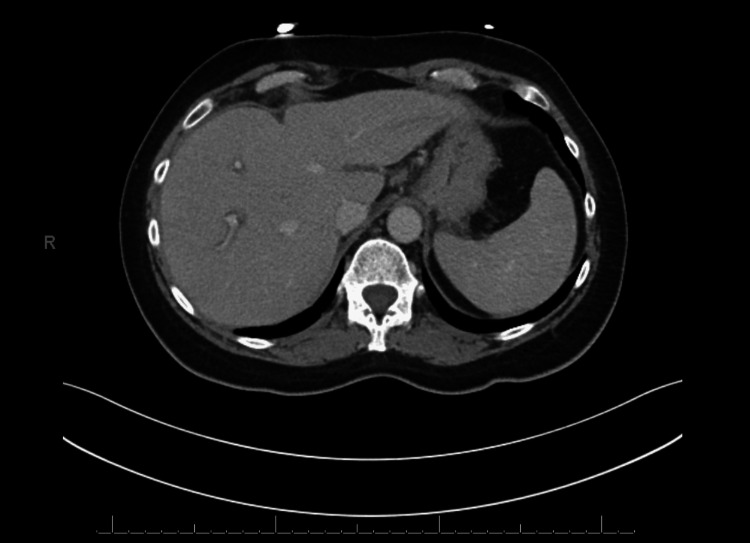
Computed tomography of the abdomen and pelvis without acute hepatobiliary abnormality Axial computed tomography image of the abdomen and pelvis demonstrating no acute hepatic abnormality and no biliary ductal dilatation, supporting the absence of an obstructive or structural cause of transaminitis.

**Figure 2:**
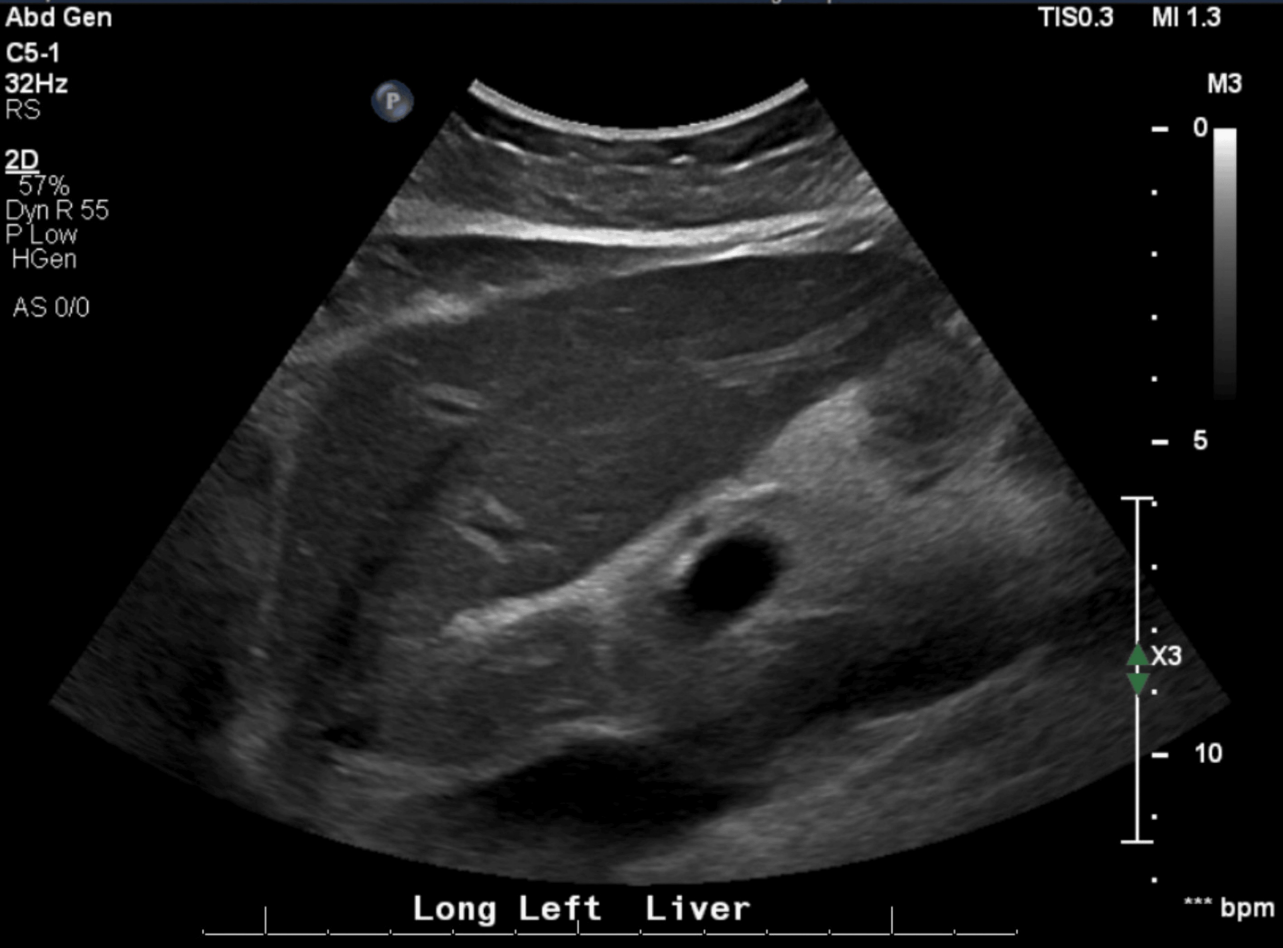
Right upper quadrant ultrasound demonstrating no biliary obstruction Right upper quadrant ultrasound image demonstrating no gallstones, no sonographic evidence of acute cholecystitis, and no intrahepatic or extrahepatic biliary ductal dilatation, supporting a non-obstructive etiology for the patient’s transaminitis.

Due to worsening symptoms, she presented to the emergency department at Tower Health Reading Hospital for further evaluation and was subsequently admitted for further workup. Laboratory testing revealed marked transaminitis. Given the severity of liver enzyme elevation, a comprehensive evaluation was undertaken. Computed tomography (CT) of the abdomen and pelvis showed no acute hepatic abnormality or biliary ductal dilatation (Figure 1), and right upper quadrant ultrasound demonstrated no sonographic evidence of acute cholecystitis or biliary obstruction (Figure 2). Serologic testing for autoimmune hepatitis, including anti-smooth muscle antibody, was negative. Immunoglobulin levels, including IgG, IgM, and IgA, were within normal limits. The hepatitis panel was negative. Liver function tests (LFTs) continued to rise, peaking in the 800 U/L range. Using the initial laboratory values, the R value was calculated as $R = \left(\frac{\mathrm{ALT}/\text{ULN ALT}}{\mathrm{ALP}/\text{ULN ALP}}\right) = \left(\frac{639/56}{104/114}\right) = 12.5$, consistent with a hepatocellular pattern of liver injury. Given persistent transaminitis and concern for DILI versus autoimmune hepatitis, a liver biopsy was performed (laboratory results are provided in Tables 1-4).

**Table 1:** Complete blood count at time of presentation

Parameter	Patient value	Reference range	Units
White blood cells	8.3	4.8-10.8	×10³/μL
Red blood cells	4.35	4.2-5.4	×10⁶/μL
Hemoglobin	12.8	12.0-16.00	g/dL
Hematocrit	36.8	36-46	%
Mean corpuscular volume (MCV)	84.6	80-99	fL
Mean corpuscular hemoglobin (MCH)	29.4	27-34	pg
Mean corpuscular hemoglobin concentration	34.8	31-37	g/dL
Red cell distribution width (RDW)	13.2	11-16	%
Platelets	282	130-400	×10³/μL

**Table 2:** Comprehensive metabolic panel ALT, alanine aminotransferase; AST, aspartate aminotransferase; BUN, blood urea nitrogen; HCO₃, bicarbonate; SGOT, serum glutamic-oxaloacetic transaminase; SGPT, serum glutamic-pyruvic transaminase.

Test	Result	Reference range	Units
Sodium	136	135-145	mmol/L
Potassium	3.3	3.5-5.0	mmol/L
Chloride	100	98-106	mmol/L
HCO_3_	26.3	22-29	mmol/L
Anion gap	10	8-16	mmol/L
BUN	17	7-20	mg/dL
Creatinine	0.83	0.6-1.3	mg/dL
Glucose	117	70-99	mg/dL
Calcium	9.2	8.5-10.5	mg/dL
AST (SGOT)	525	10-40	U/L
ALT (SGPT)	639	7-56	U/L
Direct bilirubin	1.0	0.0-0.3	mg/dL
Total bilirubin	2.1	0.1-1.2	mg/dL
Alkaline phosphatase	104	46-114	U/L
Albumin	2.7	3.4-5	g/dl

**Table 3:** Trend LFT on hospital course ALT, alanine aminotransferase; AST, aspartate aminotransferase; LFT, liver function test.

Parameter	Day 1	Day 2	Day 3	Day 4	Day 5	Day 6	Day 7	Day 8
AST (U/L)	525	489	568	645	712	793	747	650
ALT (U/L)	639	603	660	713	746	810	779	728
Direct bilirubin (mg/dL)	1.0	0.8	1.0	0.8	0.8	0.8	0.7	0.6
Total bilirubin (mg/dL)	2.1	1.8	2.1	1.8	1.9	1.6	1.5	1.3
Alkaline phosphatase (U/L)	104	88	85	86	91	87	88	88

**Table 4:** Additional laboratory tests ANA, antinuclear antibody.

Test	Result	Reference range	Units
International normalized ratio	1.3	0.9-1.1	_
Prothrombin time	15.7	11.7-14.5	sec
Ferritin	875	7-271	ng/mL
Hepatitis B surface antigen (HBsAg)	Non-reactive	Non-reactive	_
Hepatitis B core IgM antibody (HBc IgM)	Non-reactive	Non-reactive	_
Hepatitis A IgM (HAV IgM)	Non-reactive	Non-reactive	_
Hepatitis C antibody (HCV Ab)	Non-reactive	Non-reactive	_
ANA screen	Negative	Negative	_
Actin (smooth muscle) antibody IgG	<20	<20: negative; ≥20: positive	U
Ceruloplasmin	41	14-48	mg/dL
Mitochondrial (M2) antibody, IgG	≤20.0	≤20.0: negative; 20.1-24.9: equivocal; ≥25.0: positive	U
Immunoglobulin G (IgG)	1093	600-1640	mg/dL
Immunoglobulin A (IgA)	160	47-310	mg/dL
Immunoglobulin M (IgM)	180	50-300	mg/dL

**Figure 3:**
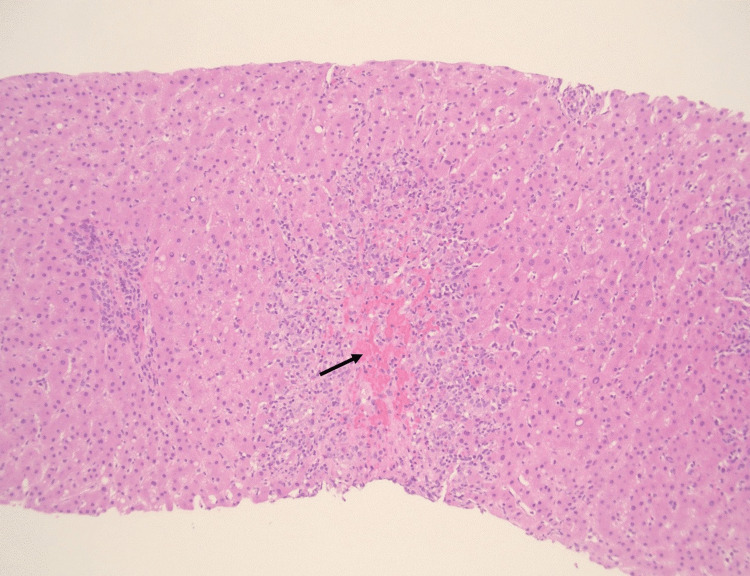
Low-power liver biopsy histology (hematoxylin and eosin) Low-power hematoxylin and eosin-stained liver biopsy section demonstrating centrilobular (zone 3) hepatocellular necrosis (arrow) with surrounding inflammatory infiltrates, consistent with drug-induced liver injury (original magnification ×10).

Histologic examination of the liver biopsy demonstrated portal tracts containing a sparse mixed inflammatory infiltrate composed predominantly of lymphocytes. Plasma cells were not prominent. Interlobular bile ducts appeared intact. There was striking centrilobular (zone 3) necrosis with associated inflammation, including macrophages, lymphocytes, and eosinophils (Figure 3). 

**Figure 4:**
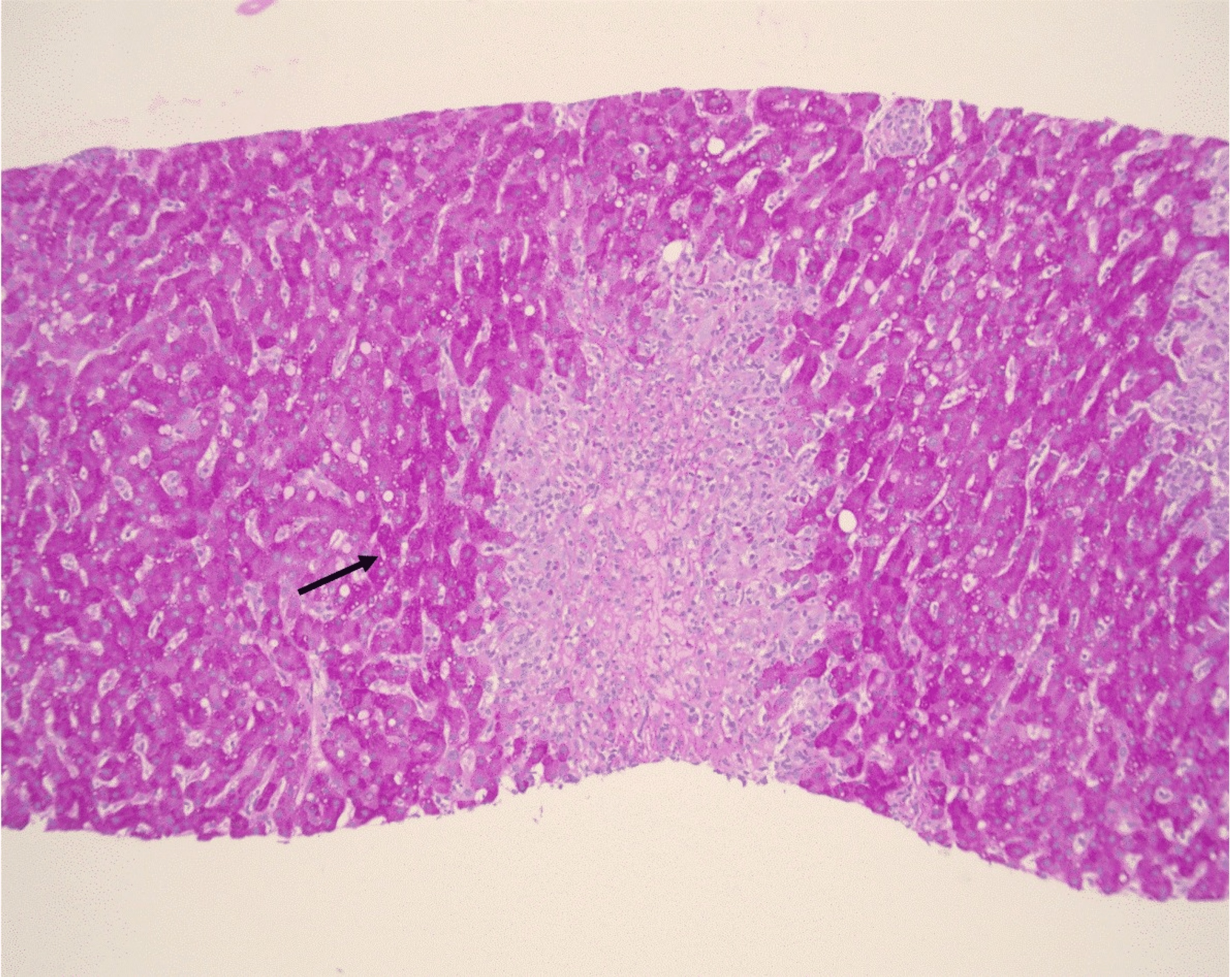
PAS-stained liver biopsy section Periodic acid-Schiff (PAS)-stained liver biopsy section demonstrating preserved cytoplasmic glycogen within viable hepatocytes (arrow) and absence of cytoplasmic inclusions, supporting drug-induced liver injury rather than a metabolic storage disorder (original magnification ×10).

Trichrome staining highlighted areas of necrosis without evidence of increased background fibrosis. Reticulin staining showed no significant fibrosis. Iron staining demonstrated a mild increase in iron, predominantly within macrophages. Periodic acid-Schiff with diastase (PAS-D) staining revealed no inclusions, while PAS staining demonstrated abundant glycogen (Figure 4).

Overall, the histopathologic findings were most consistent with DILI. The centrilobular inflammation and necrosis pattern, in conjunction with the absence of plasma cells and negative autoimmune serologies, argued against a diagnosis of autoimmune hepatitis.

Selpercatinib had been initially initiated at a dose of 160 mg twice daily. Due to concern for drug-induced hepatotoxicity in the setting of significant transaminitis and supportive biopsy findings, the medication was discontinued. Following cessation of therapy, the patient’s LFTs demonstrated progressive improvement.

Over the subsequent months, aminotransferase levels continued to decline, ultimately decreasing to below 100 U/L (reference ranges: aspartate aminotransferase: 10-40 U/L and ALT: 7-56 U/L) approximately four months after discontinuation. Given clinical stability and oncologic considerations, selpercatinib was reintroduced at a reduced dose of 40 mg daily. On follow-up monitoring, LFTs remained stable without recurrence of severe transaminitis.

## Discussion

Selpercatinib is a highly selective RET kinase inhibitor approved for the treatment of RET fusion-positive non-small cell lung cancer and RET-altered thyroid malignancies based on results from the LIBRETTO-001 study. In a final safety and efficacy analysis of LIBRETTO-001 published in the Journal of Clinical Oncology, selpercatinib demonstrated durable clinical responses with a generally manageable safety profile. However, elevated aminotransferases were among the most frequently reported laboratory abnormalities, with grade ≥3 transaminase elevations occurring in a subset of treated patients. These findings establish hepatotoxicity as a recognized adverse effect of selpercatinib therapy [2].

The precise mechanism of selpercatinib-associated hepatotoxicity remains incompletely understood. According to the National Institute of Diabetes and Digestive and Kidney Diseases LiverTox database, serum aminotransferase elevations during selpercatinib therapy are typically hepatocellular in pattern and often improve with dose interruption or reduction, suggesting a component of direct, dose-related hepatocellular injury rather than a classic idiosyncratic immune-mediated reaction. Selpercatinib undergoes hepatic metabolism primarily via the cytochrome P450 system, predominantly *CYP3A4*, and is therefore susceptible to pharmacokinetic interactions with CYP3A inhibitors or inducers. This metabolic pathway may contribute to hepatocellular stress through the accumulation of reactive metabolites or altered drug clearance in susceptible individuals [1].

To our knowledge, only one biopsy-confirmed case of selpercatinib-induced hepatotoxicity has been published in the peer-reviewed literature [4]. In that report, liver biopsy demonstrated portal-based inflammation with interface activity, lobular disarray, bile duct injury, and patchy cholestasis. In contrast, our case demonstrated striking centrilobular (zone 3) necrosis with associated lymphocytes, macrophages, and eosinophils, without prominent plasma cells or significant fibrosis. Using standard criteria outlined by the European Association for the Study of the Liver clinical practice guidelines for DILI, this pattern supports hepatocellular injury [3]. The temporal association between drug initiation and enzyme elevation, along with improvement following dose interruption (or discontinuation), further strengthens the likelihood of selpercatinib-induced liver injury. Using the updated Roussel Uclaf Causality Assessment Method (RUCAM), this case yields an approximate score in the possible-to-probable range (approximately 5-7), further supporting selpercatinib as the likely cause of hepatocellular DILI. However, this estimate is limited by the retrospective nature of the assessment and incomplete availability of some alternative-cause testing parameters [5].

Although post-marketing pharmacovigilance databases such as the FDA Adverse Event Reporting System (FAERS) contain multiple reports of abnormal hepatic function and transaminase elevations associated with selpercatinib, these entries represent spontaneous adverse event submissions rather than peer-reviewed case reports with detailed clinical evaluation, exclusion of competing etiologies, or histologic confirmation [6]. Consequently, while FAERS data suggest a safety signal, they do not provide the clinicopathologic correlation necessary to fully characterize the pattern of injury. Well-documented biopsy-proven cases, therefore, remain scarce.

The primary diagnostic consideration in this case was autoimmune hepatitis or immune-mediated hepatitis. However, several clinical and histologic features argued against these etiologies. The patient had negative autoimmune serologies, including anti-smooth muscle antibody, and normal immunoglobulin levels. Histologically, the biopsy lacked a plasma cell-rich infiltrate and did not demonstrate prominent interface hepatitis, findings typically observed in autoimmune hepatitis. Instead, the presence of marked centrilobular necrosis with eosinophilic inflammation favored a diagnosis of DILI. The temporal relationship between selpercatinib initiation and onset of transaminitis, along with progressive biochemical improvement following drug discontinuation and stability after dose reduction, further supports a causal association.

This case highlights the importance of recognizing selpercatinib as a potential cause of clinically significant hepatocellular injury. Liver biopsy may play a critical role in distinguishing DILI from autoimmune or immune-mediated hepatitis in oncology patients presenting with severe transaminitis. Reporting additional well-characterized cases will help better define the spectrum and mechanisms of hepatic injury associated with selective RET inhibition.

## Conclusions

Selpercatinib can cause clinically significant DILI with a hepatocellular pattern and characteristic histopathologic findings. Liver biopsy plays a critical role in differentiating DILI from autoimmune hepatitis in complex oncology patients. Early recognition of this complication is essential and requires careful monitoring of LFTs during treatment, particularly within the first few weeks of therapy when hepatotoxicity may emerge. Clinicians should maintain a high index of suspicion in patients who develop unexplained transaminitis or compatible symptoms such as fatigue, nausea, or right upper quadrant pain after initiating selpercatinib. Prompt evaluation, including exclusion of viral and autoimmune etiologies, calculation of the R value to determine the pattern of liver injury, and early drug interruption when indicated, may help prevent progression to severe liver injury and improve clinical outcomes. Awareness of this potential adverse effect is essential for prompt diagnosis and management.
